# Environmental pollution and economic growth: Evidence of SO_2_ emissions and GDP in China

**DOI:** 10.3389/fpubh.2022.930780

**Published:** 2022-11-10

**Authors:** Chao Yan, Huixuan Li, Zhigang Li

**Affiliations:** ^1^Center for Quantitative Economics, Jilin University, Changchun, China; ^2^Faculty of Professional Finance and Accountancy, Shanghai Business School, Shanghai, China

**Keywords:** environmental pollution, economic growth, MS (M)–VAR (p) model, SO_2_ emissions, GDP

## Abstract

This study explores the inherent linkage mechanism between environmental pollution and economic growth using a non-linear MS (M)–VAR (p) model. The results indicate that, first, the growth rates of China's gross domestic product (GDP) and SO_2_ emissions are in a state of significant inertia. Second, when the system was in a medium-growth regime, the growth rates of SO_2_ emissions and GDP had a positive correlation, characterized by lower probability and weaker durability. Third, when the system was in a high- or low-growth regime, their growth rates were negatively correlated, characterized by higher probability and stronger durability. Overall, economic growth increases environmental pollution emissions, which intensifies as well as inhibits economic growth. The correlation and sustainability of SO_2_ emissions and GDP are closely related to the regional status of the entire system. This study is helpful in analyzing the reasons for the nonlinear linkage mechanism between environmental pollution and economic growth.

## Introduction

Nature is the foundation of human existence and development. China's economy has developed sustainably and steadily, but the nation's ecological environment has been severely damaged. The carrying capacity of the environment has been reached or approached, and the problems of unbalanced, uncoordinated, and unsustainable development are still prominent. Additionally, with rapid economic and social development, the need for a clean environment has become increasingly urgent. In the face of this situation, the ecological environment quality problem has become a bottleneck in building an overall well-developed society. In the context of increasing downward pressure on the economy, it is particularly significant to balance economic development with environmental protection. It is not only crucial to reduce pollutant emissions and improve environmental quality but also achieve stable economic development. Under these conditions, how can we improve the quality of the ecological environment while maintaining steady economic growth? What is the relationship between economic growth and environmental pollution? Many scholars have studied the relationship between the two (1–3).

Based on the annual data on China's SO_2_ emissions and gross domestic product (GDP), this study constructs a two factor “environmental pollution and economic growth” system (SO_2t_-GDP_t_ system), which includes the SO_2_ emissions growth rate and the GDP growth rate. Analyzed via a non-linear MS (M)–VAR (P) model, the system is captured and depicted as a “low-growth regime” and a “medium-growth regime.” Additionally, we address the issue of the multi-stage complex dynamic change process of a “fast-growth regime,” that is, when is the system in a fast-growth regime? When will it enter a medium-growth regime and when will it fall back into a low-growth regime? How likely is the system to change interactively during different growth stages? Does the non-linear linkage mechanism between environmental pollution and economic growth remain unchanged at different growth stages? Addressing these questions scientifically will facilitate the identification of the transition points of the SO_2t_-GDP_t_ system between low-growth, medium-growth, and fast-growth regimes.

## Literature review

Economic sustainability depends on adequate production, distribution, and consumption, whereas environmental sustainability is determined by judicious use of natural resources such as water, land, air, and soil (4). Research on the relationship between economic development and environmental pollution can be traced back to the emergence of the growth limit theory, which holds that with the increase in industrial output, the consumption of natural resources, accumulation of waste, and concentration of pollutants will increase, and environmental quality will increasingly deteriorate. Additionally, excessive consumption of natural resources eventually restricts economic activities (5). However, in the 1980s, the sustainable development proposal that “economic growth will not necessarily damage the environment” prompted scholars to further explore the relationship between environmental pollution and economic growth. Cai et al. (6) found that two of the six sustainable development values—tolerance and solidarity—have a beneficial impact on a country's economic growth. Furthermore, the concept of the “Environmental Kuznets Curve” (EKC), which is also consistent with the concept of sustainable development, has been proposed. According to this idea, in the early stage of economic development, environmental pollution will continue to increase with economic development until economic growth reaches a “turning point.” Subsequently, environmental pollution will show a downward trend and environmental quality will improve; in short, there is an inverted U-shaped relationship between environmental pollution and income level (7, 8). Subsequently, much research has been conducted on the EKC.

To determine the reality of the EKC, the relationship between environmental pollution and economic growth was studied using the fixed effects model and the autoregressive distribution lag model. These results support the EKC hypothesis (1, 9). Other research results based on a simultaneous equation panel regression model have also confirmed the validity of the EKC hypothesis (10). One study, using the Panyang Lake Basin as the research object and linear and curve models, found that the relationship between environmental pollution and economic growth had EKC characteristics (11, 12). Research using the static panel data model also supports the EKC hypothesis (2). However, a few scholars, based on empirical research, have questioned the “inverted U” EKC. For example, one study found a monotone positive correlation between SO_2_ emissions, CO_2_ emissions, and per capita income under the control of a time effect (13). Murshed et al. (14) investigated the long-term association between economic globalization and energy-production-based CO2 emissions in Argentina from 1971 to 2016 using the ARDL model, and found that economic globalization reduces CO2 emissions associated with energy production and validates the EKC hypothesis. Other researchers using the fixed effects model believe that the EKC hypothesis is effective in middle- and high-income countries but ineffective in low- and middle-income countries (15). According to a co-integration test, the relationship between industrial solid waste discharge and economic growth has EKC characteristics, whereas the relationship between industrial wastewater, industrial waste gas discharge, and economic growth does not conform to EKC characteristics (16). A few studies have discussed the relationship between economic growth and carbon dioxide emissions and found that the short- and long-run parameters of the estimated vector autoregressive models are unstable (3).

As the EKC describes only the impact of economic growth on environmental pollution and does not explain the adverse effects of environmental pollution on economic growth, a co-integration test method was used, which indicated a two-way causal relationship between environmental pollution and economic growth (17). Studies based on the dynamic simultaneous equation model have found that an increase in pollutant emissions reduces the production capacity of a country (18). Using the Bayesian vector autoregression (VAR) model, owing to the role of a feedback mechanism, it was found that environmental quality optimization in the Gansu Province is conducive to sustainable economic development (19). Using a panel VAR model, it was found that environmental pollution resulting from economic growth may have a lag effect on the reverse effect of environmental pollution (20). Examples from the Shanxi Province and Nanjing City suggest that environmental pollution and economic growth have a two-way impact mechanism (21). A few studies have applied a dynamic simultaneous equation model to demonstrate that environmental pollution can promote economic growth, and economic growth can aggravate environmental pollution (22).

Research from the perspective of the “open economy” to explore the relationship between environmental pollution and economic growth has produced conflicting conclusions. For example, Singhania and Saini (23) believe that foreign direct investment (FDI) exploits the lack of mandatory statements on environmental disclosures to degrade the environment. They contend that the absence of strict laws to regulate environmental reporting of FDI contributes to its negative effects on the environment. A few researchers have indicated that, under the influence of technology and diffusion of FDI, the production level of developing countries will improve and pollutant emissions will reduce (24). Research on Southeast Asian Allies has shown that strict environmental regulations directly restrict FDI and ultimately inhibit economic growth (25). FDI and R&D investment have a substantial effect on energy-environmental performance, whereas power structure has a negative impact (26). FDI may not be the best source of economic growth in the long run, but its technology demonstration and spillover effects are conducive to China's environmental improvement (27). One study considered China's manufacturing industry as the research object and found that FDI can significantly improve environmental protection capacity (28). Conversely, it was also found that foreign trade in India promotes economic growth at the expense of the environment (29). Sometimes, international trade can reduce the pollution levels of developed countries and exacerbate the environmental pollution level of developing countries (30). The structural, scale, and technological effects of foreign trade have been found to increase China's environmental pollution (31). While governments and enterprises are vigorously increasing the level of foreign investment, they must pay equal attention to economic growth and public health. The level of industrial agglomeration should match the level of foreign investment to give full play to the positive improvement effect of industrial agglomeration on environmental pollution and realize the coordinated development of the regional economy, the environment, and population health (32).

This study contributes to the current scholarly literature. First, most domestic scholars have used inter-provincial panel data or data from one province to explore the relationship between environmental pollution and economic growth in China; few studies have been conducted at the macro level based on time-series data. Second, many scholars have built econometric models based on the idea of EKC fitting to study the relationship between environmental pollution and economic growth in China. However, these econometric models consider only the one-way impact of the economy on the environment, ignoring the effects of the environment on the economy, which has resulted in an endogenous deviation (33). Therefore, this study explores the relationship between environmental pollution and economic growth in China using the VAR model, which measures a two-way mechanism between variables (34, 35). Finally, there are many disadvantages to using linear measurement methods, such as the VAR model, to study time series with non-linear characteristics (36). Therefore, in this study, a non-linear Markov regime switch vector autoregression (MS-VAR) model was selected to measure the periodic characteristics of the non-linear linkage mechanism of environmental pollution and economic growth scientifically and accurately from a macro perspective (37).

## Materials and methods

### Construction of the non-linear MS (M)–VAR (p) model

Sim (38) proposed the classical linear VAR model, which is widely used to measure the dynamic relationship between multiple variables. Following Sim (38), this study first constructs a linear k-dimensional p-order VAR model, which has been extensively used to explore dynamic relationships among multiple variables:


(1) $$\begin{array}{l} y_{t}=v+A_{1}y_{t-1}+...+A_{p}y_{t-p}+u_{t} \end{array}$$

where $y_{t}=(y_{1t},...,y_{kt})\prime$represents the k-dimensional endogenous variable vector, and *t* = 1, …, *T* and *v* represent the intercept term. In this study, it is assumed that the time series of the k-dimensional endogenous variables are all stationary; that is, $y_{t-j}=L^{j}y_{t}$ and *L* represent lag operators. Simultaneously, assuming that the error term *u*_*t*_ in Equation (1) shows a normal distribution, that is, $u_{t}\tilde{\;}NID\left(0,\Sigma \right)$, Equation (1) is a linear VAR (p) model of the intercept form. Of course, Equation (1) can also be expressed as a linear VAR (p) model of the mean form:


(2) $$\begin{array}{l} y_{t}-\mu =A_{1}\left(y_{t-1}-\mu \right)+...+A_{p}\left(y_{t-p}-\mu \right)+u_{t} \end{array}$$

where μ represents the *k* × 1 dimensional mean of *y*_*t*_ and $\mu ={\left(I_{k}-\Sigma_{j=1}^{p}A_{j}\right)}^{-1}v$. However, the linear VAR (p) model represented by Equations (1) or (2) cannot accurately explore the potential non-linear characteristics of the endogenous variable time series. This research shows that the time series of the SO_2_ emission growth rate and GDP growth rate measured in this study contain significant non-linear characteristics. Therefore, this study uses the idea of Krolzig (39), and the non-linear “regime switch” factor is introduced into the linear VAR (p) model shown in Equations (1) and (2). In particular, it is assumed that the parameters of the *y*_*t*_ data generation process depend on the discrete variables *s*_*t*_, among which the M-region is characterized by *s*_*t*_, that is, *s*_*t*_ ∈ {1, …, *M*}; additionally, *s*_*t*_ follows the Markov process of traversing the M-region system, and its switch matrix form is as follows:


$$P=\left[\begin{array}{cccc} p_{11} & p_{12} & \ldots & p_{1M}\\ p_{21} & p_{22} & \ldots & p_{2M}\\ \vdots & \vdots & \ddots & \vdots \\ p_{M1} & p_{M2} & \ldots & p_{MM} \end{array}\right]$$

where switch probability *p*_*ij*_ = Pr(*s*_*t*+1_ = *j*|*s*_*t*_ = *i*), $\overset{M}{\underset{j=1}{\sum }}p_{ij}=1,\forall i,j\in \left\{1,\ldots ,M\right\}$.

Based on the “mean form” linear VAR (p) model shown in Equation (2), the following four types of “mean form” non-linear MS (M)–VAR (p) models can be constructed:

If the mean value μ contained in Equation (2) can be based on the variable parameter functions μ(*s*_*t*_) through the introduction of the regional variable *s*_*t*_, the following MSMA (M)–VAR (p) model is constructed:


(3) $$\begin{array}{l} y_{t}-\mu \left(S_{t}\right)=A_{1}\left[y_{t-1}-\mu \left(S_{t-1}\right)\right]+\cdots +A_{p}\left[y_{t-1p}-\mu \left(S_{t-p}\right)\right]\\ \;+\mu_{t},\;\mu_{t}\sim NID\left(0,\Sigma \right) \end{array}$$

$$\text{where }\mu (s)=\{\begin{array}{ll} \mu_{1}, & s_{t}=1\\ \vdots & \vdots \\ \mu_{M}, & s_{t}=M \end{array},$$

The expression of the variable parameter functions *A*_1_(*s*_*t*_), …, *A*_*p*_(*s*_*t*_), Σ(*s*_*t*_), and *v*(*s*_*t*_) mentioned in the following discussion is similar to that of μ(*s*_*t*_) and will not be discussed here.

If the regional variable *s*_*t*_ is introduced into the mean value μ and autoregressive coefficient *A*_1_, …*A*_*p*_ included in Equation (2), the MSMA (M)–VAR (p) model can be constructed based on the variable parameter function μ(*s*_*t*_) and *A*_1_(*s*_*t*_), …, *A*_*p*_(*s*_*t*_)


(4) $$\begin{array}{l} y_{t}-\mu \left(S_{t}\right)=A_{1}\left(s_{t}\right)\left[y_{t-1}-\mu \left(S_{t-1}\right)\right]+\cdots \\ \;+A_{p}\left(s_{t}\right)\left[y_{t-p}-\mu \left(S_{t-p}\right)\right]+\mu_{t},\;\mu_{t}\sim NID\left(0,\Sigma \right) \end{array}$$

If the regional variable *s*_*t*_ is introduced into the mean value μ, autoregressive coefficient *A*_1_, …*A*_*p*_, and heteroscedasticity Σ included in Equation (2), the MSMAH (M)–VAR (p) model can be constructed based on the variable parameter functions μ(*s*_*t*_), *A*_1_(*s*_*t*_), …, *A*_*p*_(*s*_*t*_), and Σ(*s*_*t*_)


(5) $$\begin{array}{l} y_{t}-\mu \left(S_{t}\right)=A_{1}\left(s_{t}\right)\left[y_{t-1}-\mu \left(S_{t-1}\right)\right]+\cdots \\ \;+A_{p}\left(s_{t}\right)\left[y_{t-p}-\mu \left(S_{t-p}\right)\right]+\mu_{t},\\ \;\mu_{t}\sim NID\left(0,\Sigma \left(s_{t}\right)\right) \end{array}$$

If the regional variable *s*_*t*_ is introduced into the mean value μ and heteroscedasticity Σ included in Equation (2), the MSMH (M)–VAR (p) model can be constructed based on the variable parameter functions μ(*s*_*t*_) and Σ(*s*_*t*_).


(6) $$\begin{array}{l} y_{t}-\mu \left(S_{t}\right)=A_{1}\left[y_{t-1}-\mu \left(S_{t-1}\right)\right]+\cdots \\ \;+A_{p}\left[y_{t-p}-\mu \left(S_{t-p}\right)\right]+\mu_{t},\;\mu_{t}\sim NID\left(0,\Sigma \left(s_{t}\right)\right) \end{array}$$

Similarly, based on the “intercept form” linear VAR (p) model shown in Equation (1), the following four types of “intercept form” non-linear MS (M)–VAR (p) models can be constructed:

If the regional variable *s*_*t*_ is introduced into the intercept term *v* included in Equation (1), the MSI (M)–VAR (p) model can be constructed based on the variable parameter function *v*(*s*_*t*_).


(7) $$\begin{array}{l} y_{t}=v\left(s_{t}\right)+A_{1}y_{t-1}+\cdots +A_{p}y_{t-p}\\ \;+\mu_{t},\;\mu_{t}\sim NID\left(0,\Sigma \right) \end{array}$$

If the regional variable *s*_*t*_ is introduced into the intercept term *v* and the autoregressive coefficient *A*_1_, … *A*_*p*_ is included in Equation (2), the MSIA (M)–VAR (p) model can be constructed based on the variable parameter functions *v*(*s*_*t*_) and *A*_1_(*s*_*t*_), …, *A*_*p*_(*s*_*t*_)


(8) $$\begin{array}{l} y_{t}=v\left(s_{t}\right)+A_{1}\left(s_{t}\right)y_{t-1}+\cdots +A_{p}\left(s_{t}\right)y_{t-p}\\ \;+\mu_{t},\;\mu_{t}\sim NID\left(0,\Sigma \right) \end{array}$$

If the regional variable *s*_*t*_ is introduced into the intercept term *v*, and the autoregressive coefficient *A*_1_, … *A*_*p*_ and heteroscedasticity Σ are included in Equation (2), the MSIAH (M)–VAR (p) model can be constructed based on the variable parameter functions *v*(*s*_*t*_), *A*_1_(*s*_*t*_), …, *A*_*p*_(*s*_*t*_), and Σ(*s*_*t*_)


(9) $$\begin{array}{l} y_{t}=v\left(s_{t}\right)+A_{1}\left(s_{t}\right)y_{t-1}+\cdots +A_{p}\left(s_{t}\right)y_{t-p}\\ \;+\mu_{t},\;\mu_{t}\sim NID\left(0,\Sigma \left(s_{t}\right)\right) \end{array}$$

If the regional variable *s*_*t*_ is introduced into the intercept term *v* and the heteroscedasticity Σ is included in Equation (2), the MSIH (M)–VAR (p) model can be constructed based on the variable parameter functions *v*(*s*_*t*_) and Σ(*s*_*t*_)


(10) $$\begin{array}{l} y_{t}=v\left(s_{t}\right)+A_{1}\left(s_{t}\right)y_{t-1}+\cdots +A_{p}\left(s_{t}\right)y_{t-p}\\ \;+\mu_{t},\;\mu_{t}\sim NID\left(0,\Sigma \left(s_{t}\right)\right) \end{array}$$

It should be emphasized that the four types of “mean form” non-linear MS (M)–VAR (p) models shown in Equations (3)–(6) are designed to specifically capture the phenomenon in which the mean value of the time series jumps to another new mean level immediately in the case of a regime switch. The four kinds of “intercept form” non-linear MS (M)–VAR (p) models shown in Equations (7)–(10) are all designed to expound the process in which the mean value of time series does not jump to another new mean level immediately, but smoothly transits to another new mean level in a certain period of time. In an empirical study, regardless of which model is finally chosen, the dimension k of the endogenous variable vector is 2, that is, $y_{t}=(\text{S}{\text{O}}_{2\text{t}},\text{GD}{\text{P}}_{\text{t}})\prime$, SO_2t_ represents the time series of the SO_2_ emission growth rate, and GDP_t_ represents the time series of the GDP growth rate.

In this study, we use Hamilton's expectation maximization (EM) algorithm and Krolzig's maximum likelihood (ML) estimation technology, and calculate the nonlinear MS (M)–VAR (p) model. Each iteration of the EM algorithm comprised two steps. The first is the calculation of the expected value (step E), that is, based on the two algorithms of filtering and smoothing, the estimated value of the parameter vector obtained by the maximization process is used to replace the unknown real value of the parameter vector. In this step, the estimated value of the smoothing probability, which can remember the historical process of the Markov chain, is generated. The second step is to calculate the maximum value. The process of value (step M), that is, the estimated value of the parameter vector, is regarded as the solution of the likelihood function under the first-order condition, and the smoothing probability obtained in the first step (Step E) is used to replace the conditional regime probability. In this iterative process, it is necessary to construct a new parameter vector such that the filtering probability and smoothing probability can be changed in the next process of calculating the expected value (Step E) and to ensure that the likelihood function value can be increased in each iteration [for the detailed estimation process, refer to Hamilton (40) and Krolzig (39)].

### Data

Following the approach of Wang and Huang (41) and Jiang and Chen (42), this study uses the annual data on SO_2_ emissions in China to depict the state of environmental pollution, and uses the annual data on China's GDP to present the trends of economic growth. The sample range is 1986–2018. The data were obtained from the China Stock Market & Accounting Research Database, “China Statistical Yearbook,” “China Environmental Statistical Yearbook,” and “China Environmental Bulletin.”^1^

**Figure 1:**
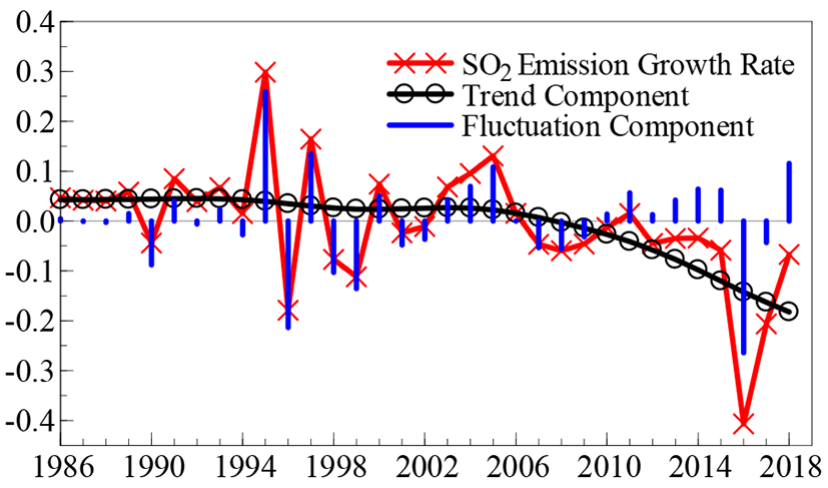
Time dynamic path of SO_2_ emission growth rate.

**Figure 2:**
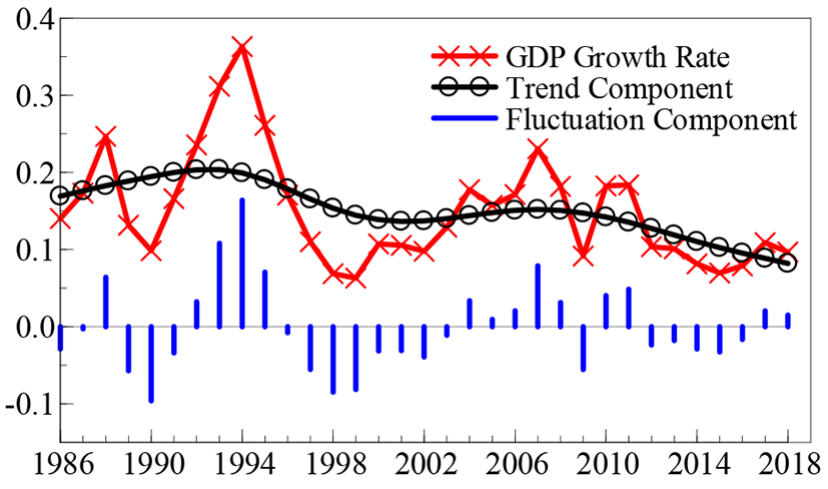
Time dynamic path of GDP growth rate.

Observation of the time dynamic path of the SO_2_ emission growth rate (SO_2t_) and the GDP growth rate (GDP_t_), outlined in Figures 1, 2, reveals that in the last 30 years, the SO_2_ emission and GDP growth rates suddenly fell back from a “peak” to a “valley” once every 10 years. Although these two growth rates show a trend of violent fluctuation, the frequency of the fluctuation in the SO_2_ emission growth rate is relatively higher. Additionally, the feedback mechanism of the effect of environmental pollution on economic growth has a certain time lag; so, these two growth rates do not show a consistent time dynamic path.

To facilitate a clearer understanding of the time dynamic change characteristics of China's SO_2_ emission and GDP growth rates, this study calculates the time dynamic path of the SO_2_ emission growth rate and GDP growth rate “trend components” and “fluctuation components,” as shown in Figures 1, 2, based on the H-P (Hodrick-Prescott) filtering technology.^2^ Among these components, the trend component reveals the long-term change trends of the SO_2_ emission and GDP growth rates, whereas the fluctuation component explains the degree of fluctuation of the two growth rates, that is, the uncertainty. Based on the trend component shown in Figure 1, the growth rate of SO_2_ emissions remained stable during the first two decades since 1986; however, in the past 13 years, the growth rate of SO_2_ emission has shown a declining trend and maintained a development level of “negative growth” for many years. In terms of fluctuation components, the growth rate of SO_2_ emissions shows weak fluctuation signs in the first 10 years since 1986; however, since the middle- and late-1990s, it shows a significant range of fluctuation clustering characteristics, and the fluctuation range reaches a maximum in 2016. The trend component presented in Figure 2 reveals that in the last 33 years, the GDP growth rate has shown two rounds of “cyclical” development trends: from a low level to a peak, and then to a valley. Over the past decade, the GDP growth rate has shown a gradual downward trend. In terms of the fluctuation component, there was a relatively significant volatility clustering phenomenon in the GDP growth rate in the first 15 years after 1986; however, in the past 15 years, the volatility of the GDP growth rate has been significantly weakened.

### Empirical results

Based on the time dynamic path initially depicted in Figures 1, 2, it is not yet possible to accurately identify the conditions under which the growth rates of SO_2_ emissions and GDP have a structural dynamic mutation. What is the potential impact of the relationship between environmental pollution and economic growth? To obtain reliable answers to these questions, this study used a non-linear MS (M)–VAR (p) model, as constructed in the second section, based on data from the SO_2_ emission growth rate time series (SO_2t_) and the GDP growth rate time series (GDP_t_) to scientifically explore the non-linear linkage mechanism between environmental pollution and economic growth in China. In the second section, when we constructed the non-linear MS (M)–VAR (p) model, it was assumed that the time series discussed met the requirement of stationarity. Thus, before conducting the measurement research, this study used ADF, PP, KPSS, and other test methods to measure the stationarity characteristics of the SO_2_ emission and GDP growth rates. The results show that the two time series were stable at the 5% significance level and a single integer at the 1% significance level.^3^ Using Akaike (AIC), Hannan and Quinn (HQ), Schwarz (SC), and other information criteria, this study determined the number of regimes of the nonlinear MS (M)–VAR (p) model to be two (*M* = 2) and three (*M* = 3). Simultaneously, when the lag order was 1–5 (*p* = 1, …, 5)^4^, the AIC, HQ, and SC values of various non-linear MS (M)–VAR (p) models shown in Equations (3)–(6) were calculated and compared. By comparison, the AIC, HQ, and SC values were calculated based on the MSIH (3) -VAR (1) model being the smallest, which shows that empirical research conducted using the MSIH (3)–VAR (1) model is the most reliable and effective when the non-linear linkage mechanism between environmental pollution and economic growth in China is analyzed.^5^ Therefore, this study conducts a systematic empirical analysis based on the MSIH (3) -VAR (1) model.

Table 1 first lists the estimated results of each parameter of the MSIH (3)–VAR (1) model calculated by the “environmental pollution and economic growth” system (hereafter referred to as the SO_2t_ − GDP_t_ system) based on the two factors of the SO_2_ emission growth rate (SO_2t_) and GDP growth rate (GDP_t_).^6^ It is found that for the dynamic regression equation of the SO_2_ emission growth rate (SO_2t_), the intercept term (−0.1740) is the smallest when it is in regime 1 (*s*_*t*_ = 1), second to largest (−0.093 1) in regime 2 (*s*_*t*_ = 2), and the largest (−0.0112) in regime 3 (*s*_*t*_ = 3). Similarly, for the dynamic regression equation of the GDP growth rate (GDP_t_), the intercept term (0.0145) is the smallest when it is in regime 1 (*s*_*t*_ = 1), second to largest (0.0168) in regime 2 (*s*_*t*_ = 2), and the largest (0.1012) in regime 3 (*s*_*t*_ = 3). Therefore, this study draws on Krolzig's idea that the growth rates of SO_2_ emissions and GDP notwithstanding, we can regard regime 1 (*s*_*t*_ = 1) as the low-growth regime, regime 2 (*s*_*t*_ = 2) as the medium-growth regime, and regime 3 (*s*_*t*_ = 3) as the fast-growth regime.

**Table 1:** Results of the MSIH (3)–VAR (1) model parameter estimations.

**Participation**	**Estimated value**		**Standard deviation**		***T***	
	**SO_2t_**	**GDP_t_**	**SO_2t_**	**GDP_t_**	**SO_2t_**	**GDP_t_**
*v*_1_	−0.1740	0.0145	0.0592	0.0073	−2.9387	1.9897
*v*_2_	−0.0931	0.0168	0.0250	0.0119	−3.7286	1.4160
*v*_3_	−0.0112	0.1012	0.0297	0.0129	−0.3756	7.8678
SO_2t-1_	0.0015	−0.0810	0.1583	0.0176	0.0092	−4.6031
GDP_t-1_	0.4491	0.6430	0.1443	0.0400	3.1117	16.0876

The significance level of the estimated values of each parameter of the MSIH (3)–VAR (1) model can be judged by means of the t-value results.

Additionally, we find a significant “inertia” characteristic in the process of GDP growth; that is, the GDP growth rate in the previous period (GD_P_t_−1_) has a significant positive effect on the current GDP growth rate (GDP_t_) (0.6430). Meanwhile, in the process of SO_2_ emission growth, there is also an inertia feature, that is, the growth rate of SO_2_ emissions in the previous period (SO_2t-1_) still has a positive impact on the growth rate of SO_2_ emissions in the current period (SO_2t_) (0.0015). Likewise, the economic structure theory (8), endogenous growth theory (46), and environmental demand theory (47) all show the impact of economic growth on environmental pollution; that is, the relationship between environmental pollution and economic growth revealed by the EKC is “inverted U-shaped.” The empirical results in Table 1 show that the GDP growth rate (GDP_t-1_) in the previous period has a significant positive effect on the current SO_2_ emission growth rate (SO_2t_) (0.4491). Alternatively, economic development in China has not yet reached a turning point where economic growth can alleviate environmental pressure, and Chen and Zhou (48) have indicated that technological progress is the main contributor to environmental performance improvement. However, China's current technological progress and economic growth structure have not yet fully exerted their effects. Thus, economic growth has a positive effect on environmental pollution. Imbalanced economic development and severe environmental quality remain significant issues that China must urgently address (49). The growth rate of SO_2_ emissions in the previous period (SO_2t-1_) had a slightly negative impact on the current GDP growth rate (GDP_t_) (−0.0810). This is because the aggravation of environmental pollution can not only reduce people's subjective well-being, affecting their quality of daily life, but also indirectly reduce employees' work efficiency, which has a negative impact on the creation of economic value. Simultaneously, enterprises with serious pollution discharge can signify a low production technology level to the capital market, thus restricting and affecting the financing ability of enterprises in the capital market. Alternatively, the aggravation of environmental pollution will increase pressure on the economic growth mode and finally restrain economic growth.

These conclusions were consistent with those of previous studies (50, 51). However, this issue must be analyzed from an empirical perspective. What is the reason for the negative effect of the growth rate of SO_2_ emissions in the previous period (SO_2t-1_) on current GDP growth rate (GDP_t_)? What type of complex non-linear dynamic relationship is involved in environmental pollution and economic growth? Does this follow certain underlying laws in non-linear dynamic correlations?

**Figure 3:**
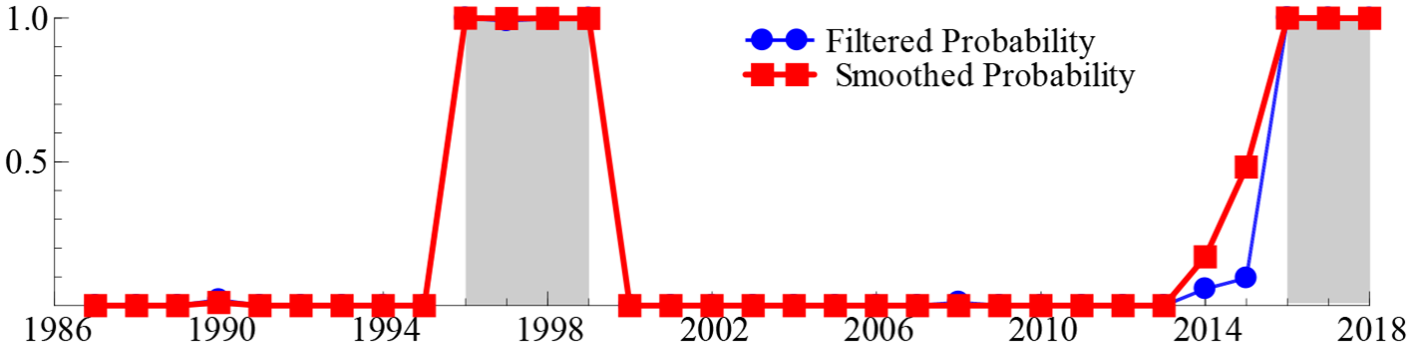
The smooth probability time dynamic path of the low-growth regime.

**Figure 4:**
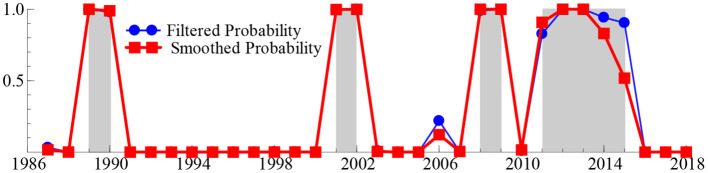
The smooth probability time dynamic path of the medium-growth regime.

**Figure 5:**
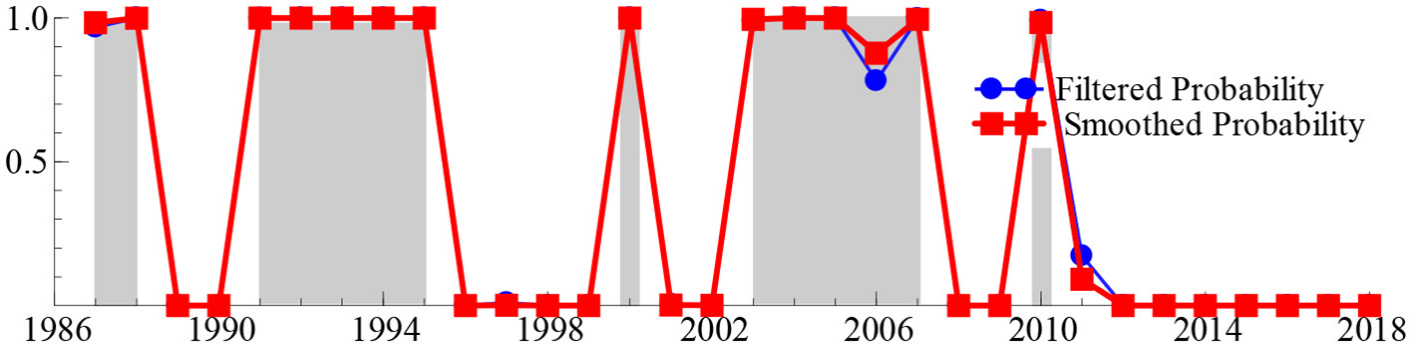
The smooth probability time dynamic path of the fast-growth regime.

To obtain reliable answers to these deep-seated problems, we next calculated the smooth probability value of the discrete value of the regime variable *s*_*t*_ based on the MSIH (3)–VAR (1) model, aiming to represent the specific regime of the SO_2t_ − GDP_t_ system over the past 33 years. When the smoothing probability value of the block variable *S*_*t*_ satisfies Pr(*s*_*t*_ = *i*|*I*_*t*_) > 0.5 and *i* = 1, 2, 3 (where *I*_*t*_ represents the information set of the past t period, the same as below), it indicates that the SO_2t_ − GDP_t_ system is in regime *i* (*i* = 1, 2, 3), and the larger the smoothing probability value, the greater the probability that the SO_2t_ − GDP_t_ system is in that regime. Table 2 lists the time intervals and smooth probability means of the SO_2t_ − GDP_t_ system in the low-growth regime (*s*_*t*_ = 1), medium-growth regime (*s*_*t*_ = 2), and high-growth regime (*s*_*t*_ = 3). Additionally, the real-time smooth probability time dynamic path of the SO_2t_ − GDP_t_ system in the low-, medium-, and fast-growth regimes is shown in Figures 3–5.

**Table 2:** Partition and smoothing probability means of the SO_2t_ − GDP_t_ system.

**Low-growth regime**		**Medium-growth regime**		**Fast-growth regime**	
**Division of**	**Smooth probability**	**Division of**	**Smooth probability**	**Division of**	**Smooth probability**
**periods**	**mean**	**periods**	**mean**	**periods**	**mean**
1996–1999	0.9992	1989–1990	0.9944	1987–1988	0.9917
2016–2018	0.9999	2001–2002	0.9986	1991–1995	0.9999
		2008–2009	1.0000	2000	1.0000
		2011–2015	0.8513	2003–2007	0.9736
				2010	0.9845

As shown in Table 2 and Figures 3–5, during 1996–1999 and 2016–2018, the “SO_2t_ − GDP_t_” system was in the low-growth regime ([Pr(*s*_*t*_ = 2|*It*) > 0.5]); in 1989–1990, 2001–2002, 2008–2009, and 2011–2015, it was in the medium-growth regime ([Pr(*s*_*t*_ = 1|*It*) > 0.5]); during 1987–1988, 1991–1995, 2000, 2003–2007, and 2010, it entered the high-growth regime ([Pr(*s*_*t*_ = 3|*It*) > 0.5]). Regardless of whether the SO_2t_ − GDP_t_ system is in the low-, medium-, or high-growth regimes, although the smooth probability value moves back and forth between different regimes, the smooth probability value in any regime is maintained at ~1.0. This not only proves that it is reasonable and reliable to identify the non-linear linkage mechanism between environmental pollution and economic growth based on the MSIH (3)–VAR (1) model in this study, but also proves that the SO_2t_ − GDP_t_ system shows the periodic dynamic mutation sign of the interaction between the low-, medium-, and high-growth regimes.

Examining the changing course of China's environmental pollution and economic growth, this study finds that from the mid-1980s to the end of the 1990s, as the governance of environmental pollution and growth of the macro-economy were in the growth stage of exploration and development, the SO_2t_ − GDP_t_ system frequently alternated between low-, medium-, and fast-growth regimes. Additionally, China's economy was affected by the Asian financial crisis, and its economic growth rate retarded. Additionally, the severity of environmental pollution and strength of national environmental governance were not significant; therefore, the SO_2t_ − GDP_t_ system was in the fast-growth regime during 1991–1995, entering the low-growth regime during 1996–1999. However, since the beginning of the 21st century, China's macroeconomic development has achieved remarkable results and stabilized. Additionally, public awareness of environmental protection has gradually improved and government's efforts in environmental governance have increased, further stabilizing and refining the SO_2t_ − GDP_t_ system. This is reflected in the fact that since the beginning of the 21st century, the frequency of alternating changes in the SO_2t_ − GDP_t_ system among the low-, medium-, and fast-growth regimes has obviously decreased. Specifically, during the early 21st century, China's economy rapidly developed; however, environmental protection still faces issues such as imperfect systems and inadequate governance. Therefore, the SO_2t_ − GDP_t_ system is primarily maintained in a rapid-growth regime. In 2006, the National Bureau of Statistics and the State Environmental Protection Administration issued the China Green GDP Accounting Report 2004, which, for the first time, made people fully aware of the harmful effects of environmental pollution on society, and even individuals. Therefore, this report provides a quantitative basis for sustainable development in China. This was precisely due to the significant strengthening of environmental pollution control by the Chinese government. Since 2007, the growth rate of SO_2_ emissions has been continuously negative for many years. The SO_2t_ − GDP_t_ system is maintained in the medium-growth regime with a relatively stable smooth probability value. From 2016 to 2020, the downward pressure on China's economy increased, and the issues of unbalanced, uncoordinated, and unsustainable development remain prominent. The demand for clean environments is becoming increasingly urgent, and it is particularly significant to balance the relationship between economic development and environmental protection. Therefore, the SO_2t_ − GDP_t_ system was transferred from a medium-growth regime to a low-growth regime during 2016–2018.

Having identified and characterized the specific regional system of the SO_2t_ − GDP_t_ system, this study further lists the correlation between the growth rates of SO_2_ emission and GDP in the low-, medium-, and high-growth regimes in Table 3. It can be seen that when the SO_2t_ − GDP_t_ system is in the low-growth regime, the growth rate of SO_2_ emissions has a negative correlation with the GDP growth rate (the correlation coefficient is −0.9364); when the SO_2t_ − GDP_t_ system enters the medium-growth regime, the growth rates of SO_2_ emissions and GDP show a positive correlation (the correlation coefficient is 0.2700); when the SO_2t_ − GDP_t_ system enters the fast-growth regime, the growth rates of SO_2_ emissions and GDP show a negative correlation again (the correlation coefficient is −0.7820). These findings provide a reasonable explanation for the issues mentioned above. Specifically, it was indicated earlier (see Table 1) that the growth rate of SO_2_ emissions in the previous period (SO_2t − 1_) had a slightly negative impact on the current GDP growth rate (GDP_t_) (−0.0810). This may be due to the system not only being in the medium-growth regime with a positive correlation between SO_2_ emission and GDP growth rates, but also entering the low-growth regime and the fast-growth regime with negative correlations between the SO_2_ emission and GDP growth rates.

**Table 3:** Estimation of correlation coefficients between the growth rates of SO_2_ emission and GDP under different regional systems.

	**Low-growth regime**		**Medium-growth regime**		**Fast-growth regime**	
	**SO_2t_**	**GDP_t_**	**SO_2t_**	**GDP_t_**	**SO_2t_**	**GDP_t_**
SO_2t_	1.0000	−0.9364	1.0000	0.2700	1.0000	−0.7820
GDP_t_	−0.9364	1.0000	0.2700	1.0000	−0.7820	1.0000

To demonstrate the conclusive and reliable basis of the above conclusion, this study further presents the block transition probability matrix and block attributes of the SO_2t_ − GDP_t_ system in Table 4. It can be seen that, for comparison, when the SO_2t_ − GDP_t_ system is in the low-growth regime, the maintenance probability (0.8270) and average duration^7^ (5.78) are the largest. In the fast-growth regime, the maintenance probability (0.6488) and average duration (2.85) are relatively small. The maintenance probability (0.5860) and average duration (2.42) were the smallest when it entered the medium-growth regime. This implies that when the SO_2t_ − GDP_t_ system enters the medium-growth regime, that is, when the growth rate of SO_2_ emissions has a positive correlation with the GDP growth rate, it is less likely and less sustainable. When the SO_2t_ − GDP_t_ system is in the low- and fast-growth regimes, that is, when the growth rate of SO_2_ emissions has a significant negative correlation with the GDP growth rate, it is more likely and more sustainable.

**Table 4:** The regime transition probability matrix and regime attributes of the SO_2t_ − GDP_t_ system.

	**Regime transition probability matrix**			**Regime attributes**	
	**Low-growth regime**	**Medium-growth regime**	**Fast-growth regime**	**Number of samples quantity**	**Average duration**
Low-growth regime	0.8270	0.0005	0.1725	7.7	5.78
Medium-growth regime	0.0942	0.5860	0.3198	10.4	2.42
Fast-growth regime	0.0687	0.2825	0.6488	13.9	2.85

## Conclusion

### Empirical findings and managerial implications

This study analyzes the non-linear linkage mechanism between environmental pollution and economic growth using inter-provincial panel data or data from one province to explore the relationship between environmental pollution and economic growth in China. Many researchers have built econometric models based on the idea of EKC fitting to investigate the relationship between environmental pollution and economic growth in China. However, this study explores the relationship between environmental pollution and economic growth in China using a VAR model, which can measure a two-way mechanism between variables. Meanwhile, there are many disadvantages of using linear measurement methods, such as the VAR model, to study time series with non-linear characteristics. In this study, Krolzig's non-linear MS-VAR model was selected to scientifically and accurately measure the periodic characteristics of the non-linear linkage mechanism of environmental pollution and economic growth from a macro perspective. This study makes three contributions to the literature.

First, we find a significant “inertia” feature in the GDP growth process, that is, the GDP growth rate in the previous period has a significant positive effect on the current GDP growth rate. There is also an inertia feature in the growth process of SO_2_ emissions, that is, the growth rate of SO_2_ emissions in the previous period still has a positive effect on the growth rate of SO_2_ emissions in the current period. The GDP growth rate in the previous period has a significant positive effect on the growth rate of SO_2_ emissions in the current period. Therefore, economic growth still has a positive effect on environmental pollution, and unbalanced economic development and severe environmental quality remain important issues that China must address. The growth rate of SO_2_ emissions in the previous period has a weak negative impact on the GDP growth rate in the current period. Therefore, the aggravation of environmental pollution will increase pressure on the mode of economic growth and ultimately restrain economic growth.

Second, we discover that the SO_2t_ − GDP_t_ system was in the low-growth regime during 1996–1999 and 2016–2018; in the medium-growth regime during 1989–1990, 2001–2002, 2008–2009, and 2011–2015; and in the high-growth regime during 1987–1988, 1991–1995, 2000, 2003–2007, and 2010. Regardless of whether the SO_2t_ − GDP_t_ system is in the low- or high-growth regimes, although the smooth probability value changes repeatedly between different zones, the smooth probability value in any zone system is maintained at approximately 1.0, which not only confirms that this approach based on the MSIH (3)–VAR (1) model to identify the non-linear linkage mechanism between environmental pollution and economic growth is more reasonable and reliable, but that the SO_2t_ − GDP_t_ system shows periodic dynamic mutation signs of interactive changes among the low-, medium-, and fast-growth regimes.

Finally, when the SO_2t_ − GDP_t_ system is in the low-growth regime, the maintenance probability and average duration are relatively large; when it is in the fast-growth regime, the maintenance probability and average duration are relatively small; when it enters the medium-growth regime, the maintenance probability and average duration are at a minimum. This implies that when the SO_2t_ − GDP_t_ system enters the medium-growth regime, that is, when there is a significant positive correlation between the growth rates of SO_2_ emission and GDP, it is less likely and less sustainable. When the SO_2t_ − GDP_t_ system is in the low- and fast-growth regimes, that is, when there is a significant negative correlation between the growth rates of SO_2_ emission and GDP, it is more likely and more sustainable.

### Limitations and directions for future research

This section discusses the limitations of this study and suggests potential directions for future research. First, the advantage of this study is the construction of an environmental pollution and economic growth system (the SO_2t_ − GDP_t_ system), which includes the SO_2_ emission growth rate (SO_2t_) and GDP growth rate (GDP_t_). Second, the nonlinear MS (M)–VAR (P) model is applied to capture and characterize the multistage complex dynamic change process of the SO_2t_ − GDP_t_ system in the low-, medium-, and fast-growth regimes, and then to identify whether the non-linear linkage mechanism of environmental pollution and economic growth has periodic characteristics in different zone systems. Thus, this study contributes to the current scholarly literature. Nevertheless, owing to data limitations, this study only analyzed the impact of environmental pollution, represented by SO_2_ emissions, on economic growth. In future research, we will explore the impacts of more diversified environmental pollution on the entire economic system and investigate whether the treatment of environmental pollution can produce high-quality economic development as a result of industrial structure upgrading.

Environmental degradation is a serious challenge in the process of human development. If a country is committed to achieving sustainable development, its first task will be to solve the environmental problems caused by development. Green development, which is the mainstream direction of China's economic and social development, plays a crucial role in leading a sustainable and steady economy. It is also the decisive force in building a moderately prosperous society. Therefore, at this stage, China should begin a reform of the environmental governance system, implement a strict environmental protection system, and improve its environmental governance capacity to ensure the stable growth of the macro-economy as well as ecological environment protection. The improvement of environmental quality requires the joint efforts of the government, enterprises, and public.

## Data availability statement

Publicly available datasets were analyzed in this study. This data can be found here: The China Stock Market and amp; Accounting Research Database, China Statistical Yearbook, the China Environmental Statistical Yearbook, and the China Environmental Bulletin.

## Author contributions

CY: visualization, methodology, writing—reviewing and editing, and conceptualization. HL: investigation, resources, data curation, formal analysis, and writing—original draft preparation. ZL: investigation, writing—reviewing and editing and, conceptualization. All authors contributed to the article and approved the submitted version.

## Funding

This work was supported by the Ministry of Education Project of Humanities and Social Sciences (No. 21YJC790136), Social Sciences Fund of Jilin Province (No. 2021B82), and Human and Social Sciences Research Project of Jilin Provincial Department of Education (No. JJKH20190210SK).

## Conflict of interest

The authors declare that the research was conducted in the absence of any commercial or financial relationships that could be construed as a potential conflict of interest.

## Publisher's note

All claims expressed in this article are solely those of the authors and do not necessarily represent those of their affiliated organizations, or those of the publisher, the editors and the reviewers. Any product that may be evaluated in this article, or claim that may be made by its manufacturer, is not guaranteed or endorsed by the publisher.

## Footnotes

^1^Presently, in the relevant statistical databases published by the “China Statistical Yearbook” (http://www.stats.gov.cn/); “China Environmental Statistics Yearbook” (http://www.stats.gov.cn/ztjc/ztsj/hjtjzl/); “China Environmental Bulletin” (https://www.mee.gov.cn/hjzl/sthjzk/zghjzkgb/) etc., we can find the annual data on China's SO_2_ emissions only from 1985 to 2018. Using these statistics, we calculated the data on China's SO_2_ emission growth rate and gross domestic product (GDP) growth rate from 1986 to 2018. For a full characterization of the dynamic correlation between air pollution and China's economic growth, other indicators of air pollution were also selected. However, as only data on nitrogen oxide emissions and smoke (powder) dust emissions for 2011–2017 were available in the official published statistical database, we constructed an MS (M) - VAR (p) model based on 2011–2017 nitrogen oxide emissions (NO), smoke (dust), dust emissions (DUST) and China's GDP statistics; the conclusions were similar to SO_2_ emissions. Therefore, this study includes only the empirical results of nonlinear linkage between air pollution and China's economic growth, represented by SO_2_ emissions. The empirical results for other indicators are listed in the Appendix.

^2^H-P filtering technology was first proposed by Hodrick and Prescott (43) in a study analyzing the characteristics of the US economic cycle after World War II and was widely used by scholars. It aims to identify and analyze the long-term trends of time series.

^3^Owing to space limitation, the specific unit root test results calculated by ADF, PP, KPSS, and other test methods are not listed in detail in this article. Interested readers can request them from the author.

^4^In the nonlinear MS (M) - VAR(P) model constructed in this study, although in theory, regime M can take any positive integer greater than zero, in the current empirical research based on the nonlinear MS (M) - VAR (P) model at home and abroad, the value of M is almost always 2 or 3. If the value of M exceeds 3, the nonlinear MS (M) - VAR (P) model loses its ability to explain the real problems. Additionally, given that the total sample number of the two time-series of the SO_2_ emission and GDP growth rates examined in this study is 33, and that the final selected model should be maximally reliable and robust, the lag order p of the nonlinear MS (M) - VAR(P) model was taken from 1 to 5 (the lag period was close to one-sixth of the total sample number).

^5^Owing to space limitation, the calculation results of the AIC, HQ, and SC values of various nonlinear MS (M) - VAR (P) models based on Akaike, Hannan and Quinn, Schwarz, and other information criteria are not listed in detail in this article. Interested readers can request them from the author.

^6^Li (44) and Wang et al. (45) also designed a specific “environment-economy” system from the perspective of a mathematical model, aiming to reflect the relationship between environment and economy.

^7^The average duration *D*[*s*_*t*_(*i*)], corresponding to the block variable *s*_*t*_, can be calculated as follows: *D*[*s*_*t*_(*i*)] = *E*[*s*_*t*_ = *i*] = 1/(1−*p*_*ii*_), *i* = 1, 2.

## References

[1] Tol RSJ. The economic effects of climate change. J Econ Perspect. (2009) 23:29–51. 10.1257/jep.23.2.29

[2] Vollebergh HRJ Melenberg B Dijkgraaf E. Identifying reduced-form relations with panel data: the case of pollution and income. J Environ Econom Manage. (2009) 58:27–42. 10.1016/j.jeem.2008.12.005

[3] Azomahou T Laisney F Van PN. Economic development and CO2 emissions: a nonparametric panel approach. J Public Econom. (2006) 90:1347–63. 10.1016/j.jpubeco.2005.09.005

[4] Krishnan R Agarwal R Bajada C Arshinder K. Redesigning a food supply chain for environmental sustainability-an analysis of resource use and recovery. J Cleaner Produc. (2020) 242:118374. 10.1016/j.jclepro.2019.118374

[5] Arrow K Dasgupta P Goulder L Daily G Ehrlich P Heal G. Are we consuming too much? J Econ Perspect. (2004) 18:147–72. 10.1257/0895330042162377

[6] Cai C Qiu R Tu YQ. Pulling off stable economic system adhering carbon emissions, urban development and sustainable development values. Front Public Health. (2022) 10:814656. 10.3389/fpubh.2022.814656 35223738 PMC8866235

[7] Harbaugh WT Levinson A Wilson DM. Reexamining the empirical evidence for an environmental kuznets curve. Rev Econom Statistics. (2002) 84:541–51. 10.1162/003465302320259538

[8] Grossman GM Krueger AB. Environmental impacts of a north american free trade agreement. Soc Sci Electr Publish. (1991) 8:223–50. 10.3386/w3914

[9] Farzin YH Bond CA. Democracy and environmental quality. J Dev Econom. (2006) 81:213–35. 10.1016/j.jdeveco.2005.04.003

[10] Omri A Daly S Rault C Chaibi A. Financial development, environmental quality, trade and economic growth: what causes what in MENA Countries. Energy Econom. (2015) 48:242–52. 10.1016/j.eneco.2015.01.008

[11] Li ZT Huang HQ Zhang MQ Wang XH. Study on the relationship between economic growth. and water environment pollution in poyang Lake Basin. Resource Sci. (2010) 32:267–73.

[12] Liu RR Deng XZ Jin Q Zheng XQ. Relationship between economic growth and nitrogen and phosphorus emissions in Poyang Lake Basin. Resource Sci. (2011) 33:2169–74.

[13] Millimet DL List JA Stengos T. The environmental kuznets curve: real progress or misspecified models? Rev Econom Statistics. (2003) 85:1038–47. 10.1162/003465303772815916

[14] Murshed MS Rashid R Ulucak V Nathaniel SP. Mitigating energy production-based carbon dioxide emissions in Argentina: the roles of renewable energy and economic globalization. Environ Sci Pollution Res. (2022) 29:16939–58. 10.1007/s11356-021-16867-y 34655033

[15] Auffhammer M Steinhauser R. Forecasting the path of US CO2 emissions using state-level information. Rev Econom Statistics. (2012) 94:172–85. 10.1162/REST_a_00152

[16] Hettige H Mani M. Wheeler, D. Industrial pollution in economic development: the. environmental kuznets curve revisited. J Dev Econom. (2000) 62:445–76. 10.1016/S0304-3878(00)00092-4

[17] Zivin JG Neidell M. Environment, health, human capital. J Economic Literature. (2013) 51:689–730. 10.1257/jel.51.3.689

[18] Fleming P Lichtenberg E Newburn DA. Evaluating impacts of agricultural cost sharing on water quality: additionality, crowding in, and slippage. J Environ Econom Manage. (2018) 92:1–19. 10.1016/j.jeem.2018.08.007

[19] Wang L Zhou D Wang Y Zha D. An empirical study of the environmental kuznets curve for environmental quality in gansu province. Ecol. Indic. (2015) 56:96–105. 10.1016/j.ecolind.2015.03.023

[20] Lisciandra M Migliardo C. An Empirical study of the impact of corruption on. Environ Performance. (2017) 68:297–318. 10.1007/s10640-016-0019-1

[21] Chen H Hu W. Determining whether trade can affect regional environmental sustainability from the perspective of environmental pollution. Sustainability. (2020) 12:1746. 10.3390/su12051746

[22] Hagens NJ. Economics for the future - beyond the superorganism. Ecol Econom. (2020) 169:106520. 10.1016/j.ecolecon.2019.106520

[23] Singhania M Saini N. Demystifying pollution haven hypothesis: role of FDI. J Business Res. (2021) 123:516–28. 10.1016/j.jbusres.2020.10.007 33100429 PMC7572317

[24] Rodrigue J Soumonni O. Deforestation, foreign demand and export dynamics in Indonesia. J Int Econom. (2014) 93:316–88. 10.1016/j.jinteco.2014.03.004

[25] Baek J. A new look at the FDI - income - energy - environment Nexus: dynamic panel data. Anal ASEAN. Energy Policy. (2016) 91:22–7. 10.1016/j.enpol.2015.12.045

[26] Yang L. Economic-environmental law guarantee of the green and sustainable development: role of health expenditure and innovation. Front Public Health. (2022) 10:910643. 10.3389/fpubh.2022.910643 35774569 PMC9238292

[27] Dasgupta S Laplante B Wang H Wheeler D. Confronting the environmental kuznets. Curve J Econom Perspect. (2002) 16:147–68. 10.1257/0895330027157

[28] Antoci A Borghesi S Russu P Ticci E. Foreign direct investments, environmental. Externalities and capital segmentation in a rural economy. Ecol Econom. (2015) 116:341–53. 10.1016/j.ecolecon.2015.04.029

[29] Tiwari AK Shahbaz M Hye QMA. The environmental kuznets curve and the role of coal consumption in India: cointegration and causality analysis in an open economy. Renew Sustain Energy Rev. (2013) 18:519–27. 10.1016/j.rser.2012.10.031

[30] Shapiro JS. Trade costs, CO2, the environment. Am Econ J. (2016) 8:220–54. 10.1257/pol.20150168

[31] He J. Pollution haven hypothesis and environmental impacts of foreign direct investment: the case of industrial emission of sulfur dioxide (SO2) in Chinese provinces. Ecol Econom. (2006) 60:228–45. 10.1016/j.ecolecon.2005.12.008

[32] Li SJ Sun B Hou DX Jin WJ Ji Y. Does industrial agglomeration or foreign direct investment matter for environment pollution of public health? Evidence From China. (2021) 9:711033. 10.3389/fpubh.2021.711033 34490192 PMC8416622

[33] Coxhead I. Skies over Beijing: economic growth and the environment in China. J Econom Literature. (2019) 57:161–79. 10.1257/jel.20171456

[34] Huang BN Hwang MJ Yang CW. Causal relationship between energy consumption and GDP growth revisited: a dynamic panel data approach. Ecol Econom. (2008) 67:41–54. 10.1016/j.ecolecon.2007.11.006

[35] Ouattara B Strobl E. The fiscal implications of hurricane strikes in the Caribbean. Ecol Econom. (2013) 85:105–15. 10.1016/j.ecolecon.2012.10.002

[36] Skalin J Terasvirta T. Another look at swedish business cycle. J Appl Econometr. (1999) 14:359–78. 10.1002/(SICI)1099-1255(199907/08)14:4andlt;359::AID-JAE517andgt;3.0.CO;2-1

[37] Leamer E Potter S. A Nonlinear Model of the Business Cycle. Manuscript, Federal Reserve Bank of New York (2003).

[38] Sims CA. Macroeconomics and reality. Econometrica. (1980) 48:1–48. 10.2307/1912017

[39] Krolzig HM. Markov Switching Vector Autoregressions: Modeling, Statistical Inference and Application to Business Cycles Analysis. University of Oxford Press (1997).

[40] Hamilton JD. Analysis of time series subject to changes in Regime. J Econometr. (1990) 45:39–70. 10.1016/0304-4076(90)90093-9

[41] Wang M Huang Y. Environmental pollution and economic growth in China. Economics. (2015) 14:557–78. 10.13821/j.cnki.ceq.2015.02.007

[42] Jiang W Chen Y. Air Pollution, foreign direct investment, and mental health: evidence from China. Front Public Health. (2022) 10:858672. 10.3389/fpubh.2022.858672 35669748 PMC9163302

[43] Hodrick RJ Prescott EC. Postwar U.S. Business cycles: an empirical investigation. J Money Credit Banking. (1997) 29:1–16. 10.2307/2953682

[44] Li XP. Analysis on the sustainability of material flow and the characteristics of circular economy material flow in environmental economic system. Resource Sci. (2008) 30:1327–35.

[45] Wang Y Zeng WH Wu SZ Jia JL. Study on environmental - economic early warning in Daxing region based on elastic coefficient. China Populat Resources Environ. (2011) 21:562–5.

[46] Stokey NL. Are there limits to growth? Int Econ Rev. (1998) 39:1–31. 10.2307/2527228

[47] Panayotou T Peterson A Sachs J. Is the environmental kuznets curve driven by structural change? CAERII Discussion Paper No. 80 (2000).

[48] Chen XH Zhou ZY. Urban environmental performance evaluation based on variable scale compensation hypothesis and its factorization. China Soft Science. (2014) 286:121–8.

[49] Li X Hu H Li MS Zhagn YJ Song JP Zhang JH. Comprehensive evaluation of china's ecological civilization and research on the coordinated development of environment, economy and society. Resource Sci. (2015) 37:1444–54.

[50] Dasgupta P Mody A Roy S Wheeler D. Environmental regulation and development: a cross - country empirical analysis. Oxford Dev Stud. (2001) 29:173–87. 10.1080/1360081012556835742576

[51] Gupta S Goldar B. Do stock markets penalize environment-friendly behavior? Evidence. (2005) 52:81–95. 10.1016/j.ecolecon.2004.06.011

