# Supplementary material for: Environmental pollution and economic growth: Evidence of SO2 emissions and GDP in China
Source: Front Public Health. 2022 Nov 10;10:930780. doi: 10.3389/fpubh.2022.930780 (PMC9684716; doi:10.3389/fpubh.2022.930780)
Supplement: Supplementary file 1 [file Table_1.pdf]

Appendix

Based on the 2011–2017 quarterly data on China's NOx emissions and its annual GDP, the current study built the "NOx emission growth rate and GDP growth rate" system (the " $NO_t - GDP_t$ " system). The results of the systematic empirical study based on the MSIH (2) -VAR (1) model are shown in Tables 1–3 and Figure 1.

Table 1

Results of the MSIH (2) - VAR (1) model parameter estimations.

Parameter	Estimated value		Standard deviation		t-value	
	NO_t	GDP_t	NO_t	GDP_t	NO_t	GDP_t
v_1	-0.0214	0.0155	0.0169	0.0025	-1.2670	6.2539
v_2	0.0052	0.0193	0.0171	0.0029	0.3046	6.5987
NO_{t-1}	0.4335	-0.0009	0.1020	0.0147	4.2498	0.6235
GDP_{t-1}	0.4893	0.1894	0.6661	0.1192	0.7347	1.5896

Notes: The significance level of the estimated values of each parameter of the MSIH (2) -VAR (1) model can be judged by the t-value results.

Table 2

Partition and smoothing probability means of the " $NO_t - GDP_t$ " system.

Low-growth regime		Fast-growth regime	
**Division of periods**	**Smoothing probability mean**	**Division of periods**	**Smoothing probability mean**
2015Q1-2016Q4	0.9998	2012Q2-2014Q4	1.0000
		2017Q1	1.0000

Table 3

The regime transition probability matrix and regime attributes of the " $NO_t - GDP_t$ " system.

	Regime transition probability matrix		Regime attributes	
	**Low-growth regime**	**Fast-growth regime**	**Number of samples quantity**	**Average duration**
**Low-growth regime**	0.8594	0.1406	8	7.11
**Fast-growth regime**	0.0841	0.9159	12	11.90

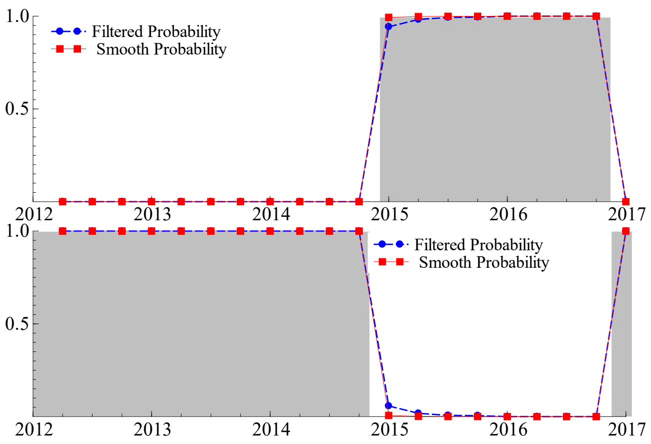

Fig. 1. The smoothing probability time dynamic path of the $NO_t - GDP_t$ system.

Based on the 2011–2017 quarterly data on smoke (powder) dust emissions and annual GDP, this study built the "Smoke (powder) dust emissions and GDP growth rate" system (the "$DUST_t - GDP_t$" system). The results of the systematic empirical study based on the MSIH (2) -VAR (1) model are shown in Tables 4–6 and Figure 2.

Table 4

Results of the MSIH (2) - VAR (1) model parameter estimations.

Parameter	Estimated value		Standard deviation		t-value	
	$DUST_t$	GDP_t	$DUST_t$	GDP_t	$DUST_t$	GDP_t
v_1	0.0689	0.0141	0.0428	0.0022	1.6113	6.4922
v_2	0.1446	0.0203	0.0535	0.0026	2.7024	7.7547
$DUST_{t-1}$	0.7133	-0.0397	0.0951	0.0047	7.4973	-8.5422
GDP_{t-1}	0.5595	0.1597	0.2714	0.1114	-2.4476	1.4333

Notes: The significance level of the estimated values of each parameter of the MSIH (2) -VAR (1) model can be judged by the t-value results.

Table 5

Partition and smoothing probability means of the "$DUST_t - GDP_t$" system.

Low-growth regime		Fast-growth regime	
**Division of periods**	**Smoothing probability mean**	**Division of periods**	**Smoothing probability mean**
2015Q1-2016Q4	1.0000	2012Q2-2014Q4	1.0000
		2017Q1	1.0000

Table 6

The regime transition probability matrix and regime attributes of the "$DUST_t - GDP_t$" system.

	Regime transition probability matrix	Regime attributes

	Low-growth regime	Fast-growth regime	Number of samples quantity	Average duration
**Low-growth regime**	0.8599	0.1401	8	7.14
**Fast-growth regime**	0.0839	0.9161	12	11.92

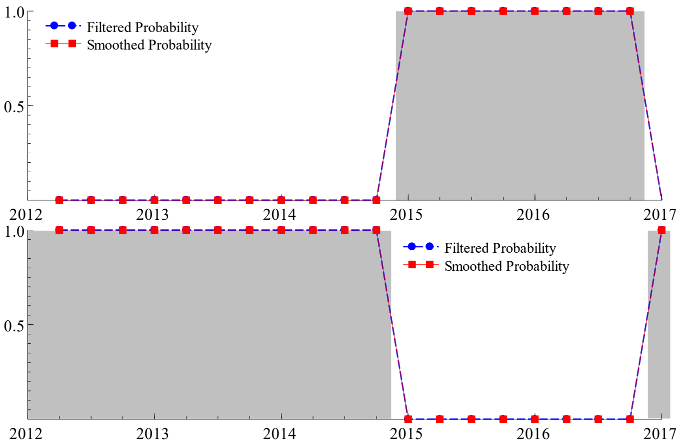

Fig. 2. The smoothing probability time dynamic path of the " $DUST_t - GDP_t$ " system.
